# ^1^H NMR fecal metabolic phenotyping of periductal fibrosis- and cholangiocarcinoma-specific metabotypes defining perturbation in gut microbial-host co-metabolism

**DOI:** 10.7717/peerj.15386

**Published:** 2023-05-09

**Authors:** Rujikorn Treeriya, Phuc N. Ho, Attapol Titapun, Poramate Klanrit, Manida Suksawat, Thanaporn Kulthawatsiri, Suphasarang Sirirattanakul, Watcharin Loilome, Nisana Namwat, Arporn Wangwiwatsin, Nittaya Chamadol, Narong Khuntikeo, Jutarop Phetcharaburanin

**Affiliations:** 1 Department of Biochemistry, Faculty of Medicine, Khon Kaen University, Khon Kaen, Thailand; 2 Department of Surgery, Faculty of Medicine, Khon Kaen University, Khon Kaen, Thailand; 3 Cholangiocarcinoma Research Institute, Faculty of Medicine, Khon Kaen University, Khon Kaen, Thailand; 4 Khon Kaen University Phenome Centre, Faculty of Medicine, Khon Kaen University, Khon Kaen, Thailand; 5 Department of Systems Biosciences and Computational Medicine, Faculty of Medicine, Khon Kaen University, Khon Kaen, Thailand; 6 Department of Radiology, Faculty of Medicine, Khon Kaen University, Khon Kaen, Thailand; 7 Center of Excellence for Innovation in Chemistry (PERCH-CIC), Faculty of Science, Khon Kaen University, Khon Kaen, Thailand

**Keywords:** Metabolomics, Periductal fibrosis, Cholangiocarcinoma, Nuclear magnetic resonance spectroscopy

## Abstract

**Background:**

The liver fluke *Opisthorchis viverrini* (OV), which subsequently inhabits the biliary system and results in periductal fibrosis (PDF), is one of the primarily causes of cholangiocarcinoma (CCA), a bile duct cancer with an exceptionally high incidence in the northeast of Thailand and other Greater Mekong Subregion (GMS) countries. Insights in fecal metabolic changes associated with PDF and CCA are required for further molecular research related to gut health and potential diagnostic biological marker development.

**Methods:**

In this study, nuclear magnetic resonance (NMR) metabolomics was applied for fecal metabolic phenotyping from 55 fecal water samples across different study groups including normal bile duct, PDF and CCA groups.

**Results:**

By using NMR spectroscopy-based metabolomics, fecal metabolic profiles of patients with CCA or PDF and of individuals with normal bile duct have been established with a total of 40 identified metabolites. Further multivariate statistical analysis and hierarchical clustering heat map have demonstrated the PDF- and CCA-specific metabotypes through various altered metabolite groups including amino acids, alcohols, amines, anaerobic glycolytic metabolites, fatty acids, microbial metabolites, sugar, TCA cycle intermediates, tryptophan catabolism substrates, and pyrimidine metabolites. Compared to the normal bile duct group, PDF individuals showed the significantly elevated relative concentrations of fecal ethanol, glycine, tyrosine, and *N*-acetylglucosamine whereas CCA patients exhibited the remarkable fecal metabolic changes that can be evident through the increased relative concentrations of fecal uracil, succinate, and 5-aminopentanoate. The prominent fecal metabolic alterations between CCA and PDF were displayed by the reduction of relative concentration of methanol observed in CCA. The metabolic alterations associated with PDF and CCA progression have been proposed with the involvement of various metabolic pathways including TCA cycle, ethanol biogenesis, hexamine pathway, methanol biogenesis, pyrimidine metabolism, and lysine metabolism. Among them, ethanol, methanol, and lysine metabolism strongly reflect the association of gut-microbial host metabolic crosstalk in PDF and/or CCA patients.

**Conclusion:**

The PDF- and CCA-associated metabotypes have been investigated displaying their distinct fecal metabolic patterns compared to that of normal bile duct group. Our study also demonstrated that the perturbation in co-metabolism of host and gut bacteria has been involved from the early step since OV infection to CCA tumorigenesis.

## Introduction

Cholangiocarcinoma (CCA), also known as bile duct cancer, is the epithelial malignancy of either intra or extra hepatic biliary tract and has been reported to be one of the most common primary liver cancers, accounting for 10–20% of all hepatic cancers (Alsaleh et al., 2019a). The exceptionally high incidence of CCA in the northeast of Thailand and other Greater Mekong Subregion (GMS) countries have caused the remarkably high morbidity and mortality rates resulting in the high socio-economic burden on patients’ families (Yongvanit, Pinlaor & Loilome, 2014). The primary cause of CCA in this GMS region is the liver fluke, *Opisthorchis viverrini* (OV) infection through traditionally- and locally-styled diets with poor sanitation that promotes the active life cycle of OV. Following infection, OV can persist in the bile duct for 20–30 years and eventually leads to cholangiocarcinogenesis (Sripa et al., 2009).

The histopathological diagnosis of periductal fibrosis from ultrasound diagnosis in CCA patients to detect early CCA cases may present as a mass or dilatation of intrahepatic duct or combination. Periductal fibrosis (PDF) is the thickening of the bile duct wall which runs parallel to the portal vein. As the parasite has inhabited the biliary system for 20–30 years, they cause the chronic inflammation of the bile ducts developing into PDF which may subsequently lead to the development of CCA (Chamadol et al., 2014). Thus, the metabolic changes and underlying mechanisms between the progression of CCA from PDF have been focused recently to improve early diagnosis as well as therapeutic approach.

Metabolomics, the latest high-throughput analysis equipped in the systems biology suite, is the quantitative and qualitative measurement of metabolites in biological specimens that has been largely employed in biomarker and molecular mechanism studies over the last decade (Nicholson, Lindon & Holmes, 1999). Recently, metabolic characterizations of CCA patient-derived specimens such as serum, urine, and tissue have been studied, thus generating several altered metabolic information in different aspects, for example, biomarker discovery and chemosensitivity (Alsaleh et al., 2020, 2019b; Padthaisong et al., 2021; Suksawat et al., 2022). Among recently reported CCA metabolomic findings, CCA urinary metabolome of different population disparities demonstrated the certain urinary metabolic fingerprints influenced by gut microbial co-host metabolism (Alsaleh et al., 2021). This metabolic interaction between host-gut microbiota has been reported to be relevant with its implication in cancer susceptibility of liver cancer, colorectal cancer, gastric cancer, and hepatobiliary cancers (Garrett, 2015). However, fecal metabolomics of CCA patients in comparison with individuals with normal bile duct and PDF have yet to be elucidated, partially due to the limitations and difficulties in sample collection. As human and other coelomate animals have symbiotic gut microbiota providing an extended genome consortium (microbiome) that interacts with the metabolism, immune systems and health of the host, it is undeniable that metabolic phenotype (metabotype) of fecal samples, thus far, gains remarkably increasing attention for its robustness in investigating not only metabolic phenoconversion but also host-gut microbial metabolic crosstalk (Waldram et al., 2009; Gratton et al., 2016). In addition, recent research has suggested that alterations in the fecal metabotype may be linked to changes in the gut microbiota (Waldram et al., 2009; Shao et al., 2017). Specifically, changes in the types and levels of metabolites in the feces may be indicative of changes in the types and levels of bacteria in the gut. Hence, the current study aimed to investigate fecal metabolome fingerprints in CCA group and non-CCA groups by using nuclear magnetic resonance (NMR) spectroscopy-based metabolomics. Accordingly, the outcomes of fecal metabotypese have potential to explain metabolic changes in gut and retrieve the effects of such consequences of OV infection, following the chronic inflammation resulted in PDF and the progression of cholangiocarcinogenesis.

## Materials and Methods

### Fecal sample collection

The fecal samples were collected from participants and patients from the same endemic area in Khon Kaen province (Khon Kaen, Thailand) where were previously recruited in Cholangiocarcinoma Screening and Care Program (CASCAP) of Khon Kaen University. Fifty-five fasting crude fecal samples were obtained from three study groups including OV-negative normal bile duct group (normal, *n* = 20), OV-positive periductal fibrosis group (PDF, *n* = 20), and OV-positive cholangiocarcinoma group (CCA, *n* = 15) without antibiotics prescribed at least 1 month prior to sample collection. Firstly, the participants were diagnosed through ultrasonography and urinary analyses and CCA patients were further confirmed by computed tomography (CT) and magnetic resonance imaging (MRI) at Srinagarind Hospital, Faculty of Medicine, Khon Kaen University, Thailand. Informed consent was obtained from all participants through Participant Informed Consent Form of Cholangiocarcinoma Research Institute. After sample collection, the fecal samples were placed on ice immediately and transferred to the laboratory. The analysis of participant demographics and clinical data including age, gender, CCA subtype, tumor morphology, and TNM stage indicative of tumor size and extent, spread to lymph nodes and metastasis was conducted using Mann-Whitney *U*-test in GraphPad Prism version 9.3.1 (350) (GraphPad Software, San Diego, CA, USA). The samples were stored at 4 °C and processed for fecal metabolite extraction within 24 h. This research was performed in accordance with relevant guidelines and regulations of Khon Kaen University Ethics Committee (KKUEC) and was approved by KKUEC for Human Research (HE571283).

### Fecal water extraction

The fecal water extraction was conducted as described elsewhere (Gratton et al., 2016). Briefly, the crude fecal samples were prepared in LC-MS grade water (CAS number 7732-18-5; Supelco, Bellefonte, PA, USA) with ratio of 1:1 of homogenized fecal sample: HPLC-grade water (g/mL). The mixture was vortexed for 20 min and further centrifuged at 4,500 rpm at 4 °C for 30 min. After the supernatant was collected, NMR buffer containing of 1.5 M KH_2_PO_4_ (CAS number 7778-77-0; Sigma-Aldrich, St. Louis, MO, USA), 2 mM NaN_3_ (CAS number 26628-22-8; Sigma-Aldrich, St. Louis, MO, USA), 1% 3-trimethylsilypropionic acid (TSP) (CAS number 29337-68-6; Cambridge Isotope Laboratories, Tewksbury, MA, USA) was added to the sample with ratio of 1:9 (mL/mL) of buffer-sample and stored at −80 °C prior further analysis.

### ^1^H NMR spectroscopic analysis

The fecal water was thawed at room temperature before centrifugation at 16,000 rpm at 4 °C for 15 min. Then, a total of 600 µL of the supernatant was transferred into 5 mm diameter NMR tube for NMR spectroscopy-based metabolomics. The operating frequency of 400 MHz (Bruker, Bremen, Germany) and detecting in standard one-dimension pulse sequence (recycle deley-90°-t1-90°-tm-°-acquisition) with t1 and to to 3 ms, tm to 10 ms, and 90° pulse to 10 µs in 64 scans were employed to acquire proton NMR spectra.

### Metabolite identification

Proton NMR spectra pre-processing (chemical shift referencing, baseline correction, and phasing) was performed in TopSpin (Bruker, Bremen, Germany). NMR spectral data were processed using MATLAB (version R2015a; MathWorks Inc., Natick, MA, USA). Further, the metabolite identification was conducted and confirmed using statistical total correlation spectroscopy (STOCSY) (Cloarec et al., 2005) in MATLAB environment and public databases including human metabolome database (HMDB) (Wishart et al., 2007, 2009, 2013, 2018) and ChenomxNMR Suite version 9.0 (Chenomx Inc., Edmonton, Canada). Data can be accessed at 10.5281/zenodo.7821106.

### Statistical analysis

After metabolite identification, the intensity of identified metabolites obtained from proton NMR spectra data was employed to perform principal component analysis (PCA) to reduce the dimensionality and raise interpretability of metabolite dataset using SIMCA-P+ version 15.0 (Umetrics Inc., Umeå, Sweden) with a unit variance scaling method. The R^2^ and Q^2^ values were collected for the goodness of fit and predictability of the model, respectively. The dataset was imported into MetaboAnalyst (Pang et al., 2021) for hierarchical clustering heat map and fold change analysis. To investigate the overview of metabolic alteartion and instinctive visualization, the hierarchical clustering and correlation heatmap of group average intensity of identified metabolites from three different groups were generated by MetaboAnalyst using Euclidean distance measure and Ward clustering method. Moreover, three pairwise fold change analyses were constructed to observe specific metabolic differences between CCA *vs*. normal, PDF *vs*. normal, and CCA *vs*. PDF with fold change threshold value of 1.5. The univariate analysis of selected metabolites was also conducted in GraphPad Prism version 9.3.1 (350) (GraphPad Software, San Diego, CA, USA) using Mann-Whitney U-test with two-tailed *p* value < 0.05.

## Results

### Demographics of participants

The study groups comprised of 55 participants including 20 individuals with PDF, 15 CCA patients and 20 control subjects with normal bile duct based on the time of first diagnosis and sampling. The diagnosis was primarily confirmed by ultrasonography for all participants and all CCA patients were further diagnosed and confirmed through computed tomography (CT) and magnetic resonance imaging (MRI) under the Cholangiocarcinoma Screening and Care Program (CASCAP) at Srinagarind Hospital, Faculty of Medicine, Khon Kaen University, Thailand. An overview of important participant demographics is summarized in Table 1. Two-tailed Mann-Whitney *U*-test showed no significant difference in age (*p* > 0.05) between these groups. There were more male than female participants in both PDF (M/F >1) and CCA (M/F >1) groups. This was perhaps expected as the higher incidence and prevalence rates of CCA in the male population. In the CCA group, most of them (46.6%) were diagnosed with perihilar CCA. Moreover, advance stage of CCA patients was presented as most cases. Typically, majority of CCA patients presented themselves at the very late stage, TNM stage III and IV (73.3%).

**Table 1:** Demographics of participants included in classification modelling and statistical analysis.

Characteristics	Non-CCA		CCA (*n* = 15)
	Normal (*n* = 20)	PDF (*n* = 20)	
Age (years)^a^	62.7 ± 12.5	58.5 ± 8.0	60.9 ± 9.0
Gender			
Male, *n* (%)	10 (50)	16 (80)	12 (80)
Female, *n* (%)	10 (50)	4 (20)	3 (20)
Male: Female^b^	1	>1	>1
CCA subtype, *n* (%)			
Intrahepatic CCA			6 (40)
Perihilar CCA			7 (46.6)
Distal CCA			1 (6.7)
Missing data			1 (6.7)
Tumour morphology, *n* (%)			
Mass forming			5 (33.3)
Intraductal			5 (33.3)
Periductal infiltrating			5 (33.4)
TNM stage, *n* (%)			
I, II			4 (24.7)
III, IV			11 (73.3)
Metastasis, *n* (%)			
Yes			5 (33.3)
No			10 (66.7)

**Notes:**

a Age values are represented as mean ± standard deviation.

b Represent as a ratio (Male:Female).

### Fecal metabolome characterization

**Figure 1:**
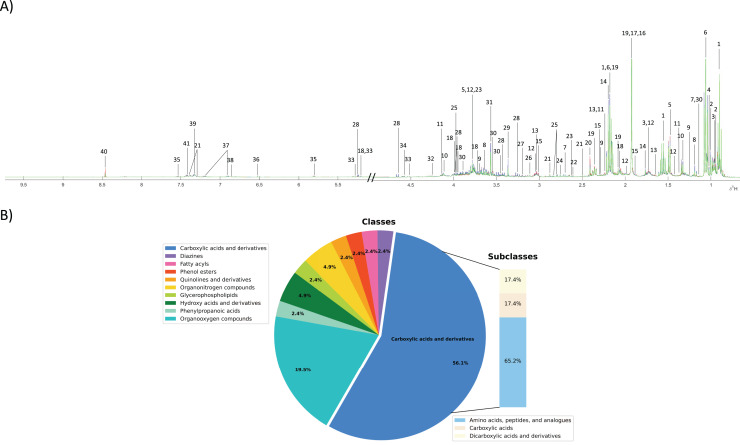
Fecal metabolic characterization using ^1^H NMR spectroscopy. (A) ^1^H NMR CPMG median spectra of fecal samples obtained from participants with normal bile duct (green), PDF (blue) and CCA (red). (B) All fecal metabolites were categorized into different chemical classes.

To investigate the fecal metabolic alteration across different groups including normal, PDF and CCA groups, 55 fecal water samples were analyzed using ^1^H NMR spectroscopy-based metabolomics with a limit of detection of approximately ≥3 μM. The spectral data were acquired and digitized into data matrix in MATLAB environment and three ^1^H NMR CPMG median spectral representatives were obtained. A total of 40 fecal metabolites were identified (Fig. 1A). A full list of identified fecal metabolites and their chemical shifts can be found in Supplementary data (Table S1). These metabolites were categorized into 10 chemical classes of which carboxylic and derivatives predominated and accounted for 56.1% that can be further sub-categorized into three chemical sub-classes including amino acids, peptides and analogues (65.2%), carboxylic acids (17.4%) and dicarboxylic acids and derivatives (17.4%) (Fig.1B). Organooxygen compounds were the second highest predominated chemical classes contained in fecal metabolites of the study groups accounted for 19.5%, followed by hydroxy acids and derivatives (4.9%), organonitrogen compounds (4.9%), and phenylpropanoic acids, glycerophospholipids, quinolines and derivatives, phenol esters, fatty acyls and diazines accounting for 2.4% each (Fig. 1B).

### PDF- and cholangiocarcinoma-associated metabotypes

**Figure 2:**
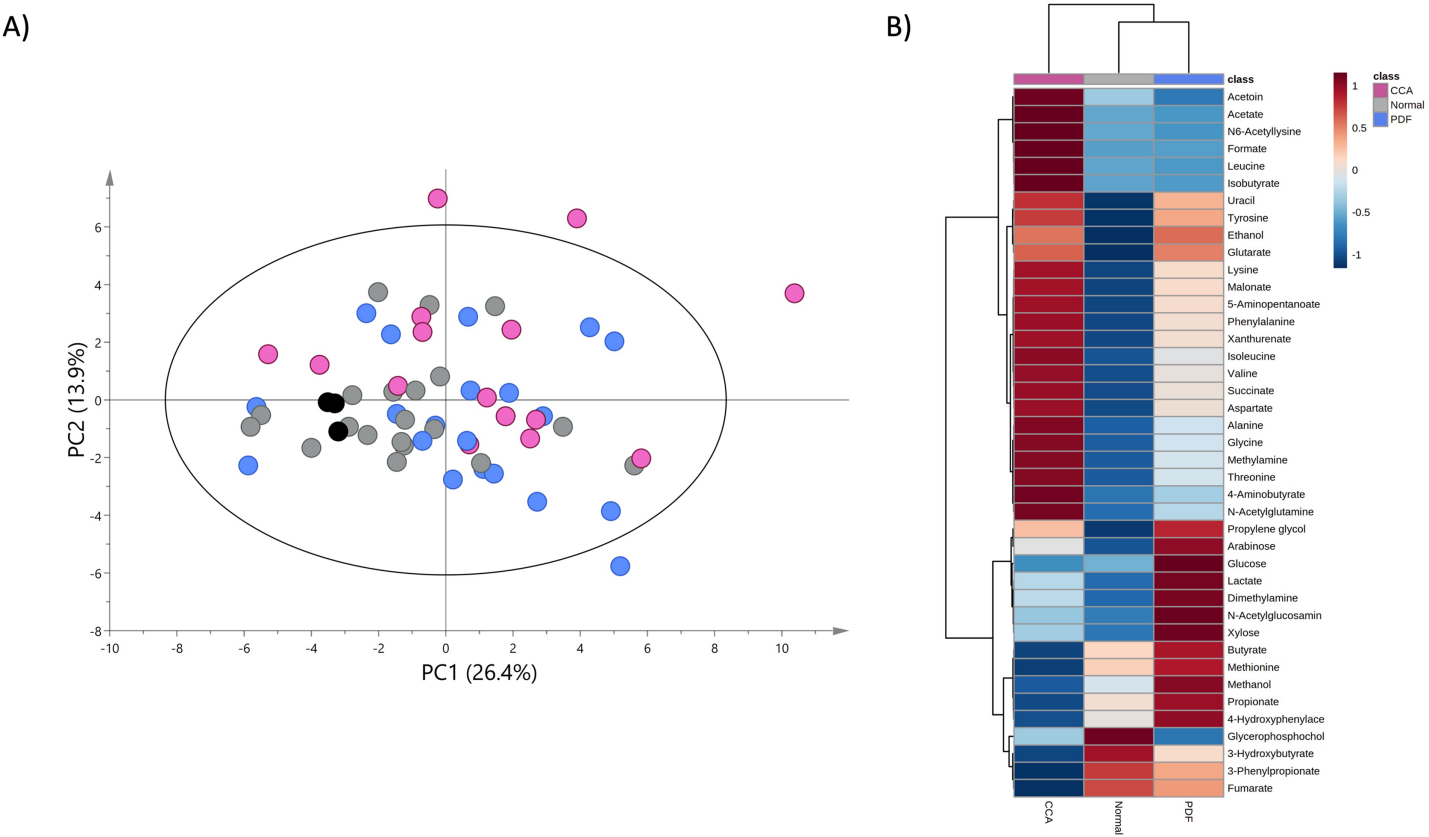
Principal component and heat map analyses of fecal metabolome data. (A) PCA scores plot of fecal metabolome data obtained from participants with normal bile duct (grey), PDF (light blue), CCA (pink) and quality control (black) using unit variance scaling. (B) Heat map was constructed using group average intensity of identified metabolites with Euclidean distance measure and Ward clustering method. Red-blue colour gradient represents z-score distribution of the group average intensity of each metabolite, with red for high peak intensity and blue for low peak intensity.

A PCA model was constructed using the entire metabolome dataset obtained from three study groups based on two principal components with pareto scaling to visualize the metabolic similarities and differences, intra- and inter-variation and outliers of the dataset. PCA scores plot with 95% confidence Hotelling’s T2 ellipse (R^2^: PC1 = 26.4%; PC2 = 13.9%; Q^2^ = 0.2) demonstrates the tight clustering of quality control (QC) samples indicating no analytical variation and high analytical precision (Fig. 2A). The trend of CCA group segregation away from PDF and normal groups was barely seen along the second principal component (PC2) with quite a large degree of intra-variation within CCA group (Fig. 2A).

To investigate the certain metabolic signatures specifically to either PDF or CCA, the relative concentrations of identified metabolites were employed for heatmap analysis using Euclidean distance measure and Ward clustering method (Fig. 2B). PDF-specific metabotype exhibited the elevated relative concentrations of sugar metabolites (*e.g*., arabinose, glucose and xylose), short-chain fatty acids (SCFAs) (*e.g*., butyrate and propionate), alcohol (*e.g*., methanol), amine metabolite (*e.g*., dimethylamine), anaerobic glycolytic metabolite (*e.g*., lactate) and microbially metabolic growth substrate (*e.g*., 4-hydroxyphenylacetate) (Fig. 2B). Interestingly, CCA-specific metabotype was revealed through the remarkable increase in the relative concentrations of the fecal amino acids (*e.g*., isoleucine, leucine, valine, alanine, lysine, aspartate, glycine, threonine, tyrosine and phenylalanine), amino fatty acids (*e.g*., 5-aminopentanoate and 4-aminobutyrate), SCFAs (*e.g*., isobutyrate, acetate), tricarboxylic acid (TCA) cycle intermediate (*e.g*., succinate), pyrimidine (*e.g*., uracil), amine metabolite (*e.g*., methylamine), microbially physiological metabolite (*e.g*., acetoin), tryptophan catabolism substrate (*e.g*., xanthurenate) and alcohol (*e.g*., ethanol) (Fig. 2B). Besides, few metabolites were observed with low relative concentrations in CCA group compared to those of PDF group (*e.g*., butyrate, methionine, methanol, propionate and 4-hydroxylphenylacetate) and of normal bile duct group (*e.g*., glycerophosphocholine, 3-hydroxybutyrate, 3-phenylpropionate and fumarate) (Fig. 2B). Overall, fecal metabolome data obtained from PDF and CCA group have displayed distinct patterns of metabotypes compared to those with normal bile duct.

**Figure 3:**
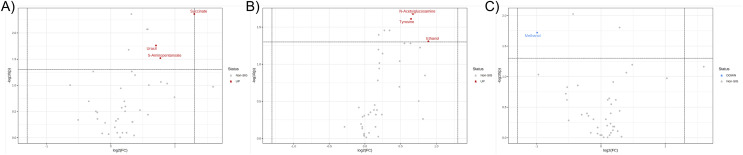
Volcano plots where the fold change analysis with threshold values of 1.5 and nonparametric test with unequal variance were performed aiming to annotate the differential metabolites in pairwise comparisons. (A) CCA and normal, (B) PDF and normal, and (C) CCA and PDF. Metabolites highlighted in either red or blue are differential metabolites that passed the fold change cut-off and *p*-value < 0.05 possessing higher concentration in either group of pairwise comparison model.

Further, the relative concentrations of identified metabolites were imported for fold change and univariate analyses to discover the significant differences in different metabotypes among three study groups. Data were presented in the volcano plots where the fold change analysis with threshold values of 1.5 and nonparametric test with unequal variance (*p*-value < 0.05) were performed aiming to annotate the differential metabolites in pairwise comparisons (Fig. 3). The CCA group demonstrated the remarkable increase in fecal concentrations of 5-aminopentanoate, succinate, and uracil (Fig. 3A) whereas PDF group has shown significantly elevated relative concentrations of fecal *N*-acetylglucosamine, tyrosine, and ethanol compared to normal group (Fig. 3B). Moreover, the differentiation between PDF and CCA groups was specified by the reduction of fecal methanol level in CCA group (Fig. 3C).

## Discussion

Cholangiocarcinoma or bile duct cancer is a malignancy that has its origin of the disease at cholangiocyte-lining cells. The inducing factors of carcinogenesis include damage caused by liver fluke, fluke secretory toxins, and host immune responses that result in chronic inflammation and hepatobiliary abnormalities. Specifically in the case of liver fluke infection, the parasite OV inhabits the biliary system for years and thus this persistent infection leads to chronic inflammation and PDF in the bile duct which can be diagnosed by ultrasonography (Chamadol et al., 2014). Importantly, PDF has been reported to be a great potential indicator for suspected CCA cases through ultrasonography from differentiation of normal and abnormal echo patterns of the bile duct (Chamadol et al., 2019). However, insights in PDF- or CCA-related gut health are required for diagnostics and prognostics of any possible gut-related complications. In this study, NMR-based metabolomics was employed to investigate the fecal metabolic changes associated with PDF and CCA to consolidate the knowledge and further our understanding in the metabolic alteration in the gut, potentially giving rise in the precaution of gut-related complications continuously induced by CCA progression.

**Figure 4:**
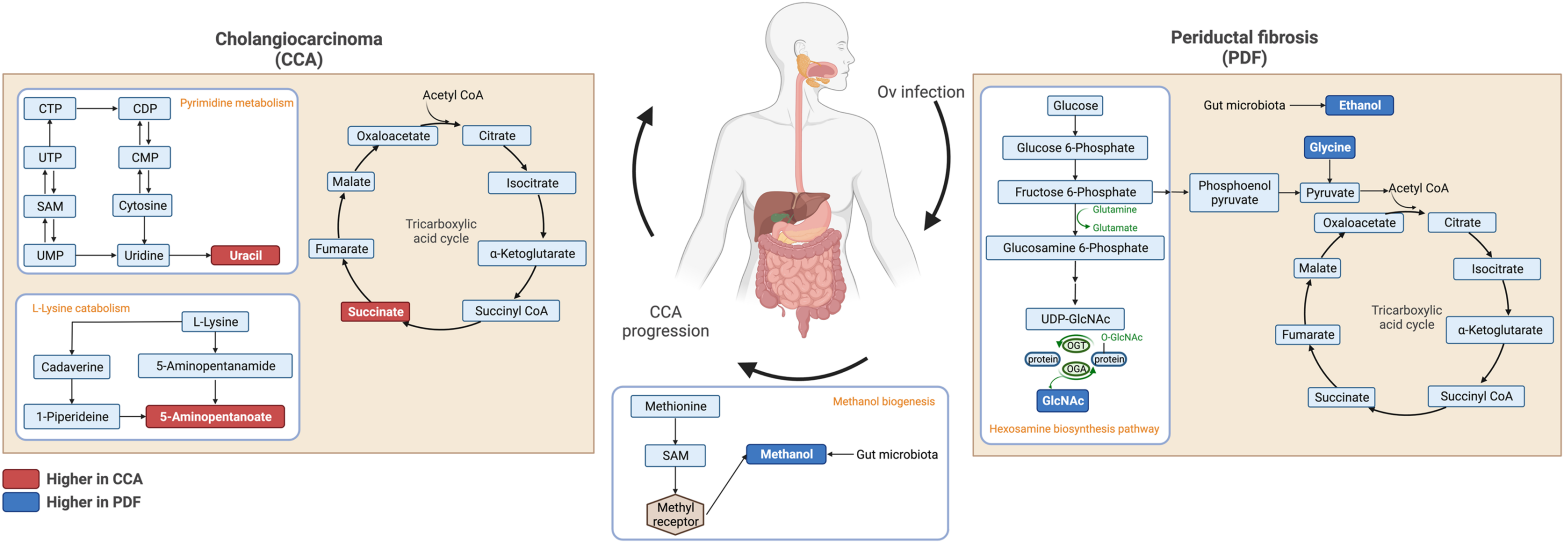
Schematic diagram of proposed metabolic alterations associated with PDF and CCA progression. This diagram was drawn using BioRender.com.

Fecal metabolic profiles of patients with CCA and PDF together with individuals with normal bile duct have been established in the current study. Our findings exhibited the distinct fingerprints of metabolic phenotypes of CCA, PDF group and normal group defining the PDF- and CCA-associated metabotypes. Participants who were diagnosed with either PDF or CCA have shown the certain metabolic alteration through various metabolite groups including amino acid, sugar, anaerobic glycolytic metabolite, alcohol, amine, TCA cycle intermediate, tryptophan catabolism substrate, pyrimidine, microbial metabolites and fatty acids (*e.g*., amino fatty acids, SCFAs). Participants diagnosed with liver fluke infection and consequent PDF have shown significant alterations in ethanol, glycine, tyrosine, and *N*-acetylglucosamine. Consequently, the development of CCA has led to remarkable metabolic changes in the gut that can be evident through elevated relative concentrations of uracil, succinate, and 5-aminopentanoate compared with normal group and when compared with PDF, reduced relative concentration of methanol was observed in CCA. Taken together, the summary of metabolic alteration associated with PDF or CCA is presented in Fig. 4. Our schematic diagram proposes the involvement of various host and host-microbial metabolic pathways that may potentially occur in the gut in parallel with the CCA progression. The chronic inflammation of the bile ducts caused by OV has shown effects on TCA cycle, ethanol biogenesis, hexamine pathway, and eventually methanol biogenesis. Tumorigenesis of CCA appeared to be highly associated with the upregulated activity of TCA cycle, pyrimidine metabolism, and lysine metabolism.

Metabolic characterizations of CCA patients have been achieved over the last few years. Sera of CCA patients were characterized and compared with a few liver morbidities including bile duct strictures and hepatocellular carcinoma (HCC) as well as in comparison to healthy control (Alsaleh et al., 2020). The previous study suggested that the altered serum metabolic profiles of the CCA group indicated inflammation, shifted energy production, and phospholipid metabolism. Moreover, urinary metabolic profile was also studied in CCA. Urinary samples of high risk of infection, OV infected, periportal fibrosis, and CCA were characterized, and the results indicated the change of energy metabolism supporting proliferation, DNA methylation, and injury of liver (Alsaleh et al., 2019b). The fecal metabolic profiles, in the current study, ultimately highlighted the perturbation in gut-microbial host co-metabolism along the progression of PDF and CCA. In the current study, we found elevated levels of fecal 5-aminopentanoate, succinate and uracil in the CCA group compared with its normal bile duct counterparts. 5-aminopentanoate is a product of lysine degradation produced by gut bacteria. Elevated levels of fecal 5-aminopentanoate may be indicative of microbiome disturbances or dysbiosis, which can be associated with a range of health conditions such as inflammatory bowel disease (IBD), irritable bowel syndrome (IBS), and colorectal cancer (Mackner et al., 2021). The significantly elevated level of fecal 5-aminopentanoate was also detected in urinary metabolic profile of laboratory animal model of *O. felineus*, another liver fluke of the Opisthorchiidae family (Kokova et al., 2017). Moreover, functional enrichment analysis of CCA tumor differentiation hub genes has defined lysine degradation as one of the ten enriched pathways indicating its critical impact related to CCA progression (Long et al., 2021). Hence, the implication of lysine catabolism in CCA tumor differentiation associated with PDF has been strengthened and could be potentially employed for further studies in the aspect of dysbiosis-induced gut inflammation. Succinate is a metabolic intermediate of the TCA cycle within the host cells and is also produced in large proportions during bacterial fermentation of dietary fiber. Elevated levels of succinate within the gut lumen have been previously reported in association with dysbiosis as well as in IBD patients and animal models of intestinal inflammation (Setoyama et al., 2003; Macias-Ceja et al., 2019) and suggest higher abundance of succinate-producing bacteria (Connors, Dawe & Van Limbergen, 2019). In addition to effects on host tissue, increased succinate levels within the intestinal lumen also change the metabolic landscape of gut microbiota communities, potentially enhancing the expansion of pathobionts that utilize succinate as a nutrient source (Kim et al., 2017). In this study, we have, for the first time, provided information and strengthened the hypothesis on the remarkable shifts in both metabolic and microbial landscapes within the intestinal lumen resulting in the potential development of intestinal inflammation in CCA that requires further investigation.

Interestingly, our findings demonstrated the elevated levels of ethanol, *N*-acetylglucosamine and tyrosine in PDF compared with normal bile duct group. Ethanol biogenesis was known to derive from either gastrointestinal or intratumoral bacteria and correlated with chemotherapeutic drug response in CCA, as shown in metabolomic study of gemcitabine- and cisplatin-sensitive groups (Suksawat et al., 2022). Importantly, endogenous ethanol has been detected in CCA tumor tissues (Suksawat et al., 2022). Thus, our results further elucidated that fecal ethanol existence is probably due to gut microbiota activity and could be initiated from PDF occurrence to persistent CCA development. *N*-acetylglucosamine is derived from the breakdown of complex carbohydrates such as chitin and mucin by intestinal microbiota through several mechanisms (Sicard et al., 2018). These bacteria possess enzymes, such as chitinases and glycosidases, that can cleave the glycosidic bonds between the sugar molecules in these complex carbohydrates, releasing *N*-acetylglucosamine (Liu et al., 2013). Another mechanism involves the *de novo* synthesis of *N*-acetylglucosamine by certain bacteria in the gut. These bacteria possess enzymes, such as glucosamine-6-phosphate synthase and *N*-acetylglucosamine-phosphate mutase, that can convert glucose-6-phosphate to *N*-acetylglucosamine-6-phosphate, which can then be dephosphorylated to form *N*-acetylglucosamine (Liu et al., 2013). In addition to *N*-acetylglucosamine producers, some gut bacteria such as *Bacteroides thetaiotaomicron* as well as some species of the genera Bacteroides, Clostridium, Lactobacillus, and Streptococcus are capable of scavenging *N*-acetylglucosamine from their environment, including the host mucosa. These bacteria possess transporters that can import *N*-acetylglucosamine into the cell, where it can be used for various functions, including energy production and cell wall synthesis (Laville et al., 2019). The ability of intestinal bacteria to either produce or utilize *N*-acetylglucosamine is an important aspect of their metabolism and plays a role in the maintenance of gut homeostasis. The amount of fecal *N*-acetylglucosamine can therefore be used as an indicator of the activity or perturbed function of the intestinal microbiota.

Another shifted gut-microbial host metabolic crosstalk detected in our fecal metabolome dataset was methanol biogenesis which potentially takes place in the intestinal lumen along the transformation from PDF to CCA. Human endogenous methanol production comes from diverse sources including methyl group donors and/or intestinal microbiota (Dorokhov et al., 2015). Despite its physiological occurrence of small level in healthy person, the alteration of endogenous methanol production was proposed to involve in the regulation of different genes related to alcoholism metabolism, neurodegenerative diseases, cardiovascular disease, and tumoral diseases (Dorokhov et al., 2015). Given the higher accumulation of methanol in PDF compared to CCA, it could display either the perturbation of specific microbial populations, in particular methanol producers, or the alteration of gene expression involved in endogenous methanol metabolism. The alteration of gut-microbiota during OV infection could be associated with elevated production of alcohol metabolites. Collectively, our findings clearly depicted the remarkable alteration in the co-metabolism of gut and/or intratumoral bacteria, and host that occurred since OV infection upon CCA tumorigenesis.

## Conclusions

In this study, NMR-based metabolomics was employed to investigate the fecal metabolic shifts in three study groups including normal bile duct, PDF and CCA groups. Our findings revealed that the perturbation of gut microbial-host co-metabolism occurred through the altered levels of microbial and host-microbial metabolites indicating the potential dysbiosis that may result from the changes in ecological niche of the gut. This could draw greater attention from physicians to the monitoring of gut health in PDF and CCA patients at the following hospital visits.

## Supplemental Information

10.7717/peerj.15386/supp-1 Supplemental Information 1 List of identified metabolites.

10.7717/peerj.15386/supp-2 Supplemental Information 2 Raw data of participants recruited in this study.

## Additional Information and Declarations

### Data Availability

The following information was supplied regarding data availability: The data is available at Zenodo, “^1^H NMR Fecal Metabolic Phenotyping of Periductal Fibrosis- and Cholangiocarcinoma-Specific Metabotypes Defining Perturbation in Gut Microbial-Host Co-Metabolism.” April 12, 2023. DOI 10.5281/zenodo.7821106.
