# Supplementary material for: 1H NMR fecal metabolic phenotyping of periductal fibrosis- and cholangiocarcinoma-specific metabotypes defining perturbation in gut microbial-host co-metabolism
Source: PeerJ. 2023 May 9;11:e15386. doi: 10.7717/peerj.15386 (PMC10178365; doi:10.7717/peerj.15386)
Supplement: Supplemental Information 1 [file peerj-11-15386-s001.docx]

**Table S1** List of identified metabolites.

| **No.** | **Metabolite** | **Chemical shift** |
| --- | --- | --- |
| 1 | Butyrate | 0.8995 (t); 1.554 (m); 2.164 (t) |
| 2 | Isoleucine | 0.9422 (t); 1.004 (d); 1.25 (m); 1.97 (m); 3.66 (d) |
| 3 | Leucine | 0.95 (d); 0.9617 (d); 1.729 (m); 2.12 (ddd); 3.72 (t) |
| 4 | Valine | 1.022 (d); 1.037 (d); 2.26 (m); 3.60 (d) |
| 5 | Alanine | 1.477 (d); 3.78 (q) |
| 6 | Propionate | 1.061 (t); 2.18 (m) |
| 7 | Isobutyrate | 1.139 (d); 2.706 (m) |
| 8 | Ethanol | 1.189 (t); 3.656 (q) |
| 9 | 3-Hydroxybutyrate | 1.244 (d); 2.302 (dd); 3.712 (m) |
| 10 | Lactate | 1.327 (d); 4.117 (q) |
| 11 | Acetoin | 1.374 (d); 2.231 (s); 4.134 (q) |
| 12 | Lysine | 1.444 (m); 1.729 (m); 1.995 (m); 2.12 (dtd); 3.033 (m); 3.784 (t) |
| 13 | 5-Aminopentanoate | 1.6467 (m); 2.237 (t); 3.022 (t) |
| 14 | Glutarate | 1.766 (m); 2.201 (t) |
| 15 | 4-Aminobutyrate | 1.907 (m); 2.302 (t); 3.021 (m) |
| 16 | Acetate | 1.92 (s) |
| 17 | *N6*-Acetyllysine | 1.38 (m); 1.42 (m); 1.55 (m); 1.58 (m); 1.89 (dtd); 1.93 (s); 2.52 (dtd); 3.17 (t); 3.75 (t) |
| 18 | *N*-Acetylglucosamine | 2.064 (s); 3.723 (d); 3.935 (d); 4.014 (dd); 4.05 (dd); 5.21 (d) |
| 19 | *N*-Acetylglutamine | 2.082 (s); 1.924 (dtd); 2.178 (m); 2.354 (dt); 4.15 (t) |
| 20 | Succinate | 2.41 (s) |
| 21 | 3-Phenylpropionate | 2.495 (t); 2.898 (t); 7.272 (t); 7.31 (d); 7.358 (t); 7.43 (m) |
| 22 | Methylamine | 2.609 (s) |
| 23 | Methionine | 2.63 (t); 2.11 (dtd); 2.143 (s); 2.19 (dtd); 3.766 (dd) |
| 24 | Dimethylamine | 2.758 (s) |
| 25 | Aspartate | 2.79 (dd); 2.658 (dd); 3.903 (dd) |
| 26 | Malonate | 3.112 (s) |
| 27 | Glycerophosphocholine | 3.2054 (s) |
| 28 | Glucose | 3.255 (dd); 3.433 (t); 3.889 (dd); 4.645 (d); 5.239 (d) |
| 29 | Methanol | 3.366 (s) |
| 30 | Propylene glycol | 3.457 (q); 1.139 (q); 3.55 (q); 3.894 (m) |
| 31 | Glycine | 3.569 (s) |
| 32 | Threonine | 1.32 (t); 4.262 (m); 3.593 (d) |
| 33 | Arabinose | 3.68 (dd); 3.81 (dd); 3.87 (dd); 4.00 (ddd); 4.517 (d); 5.21 (t); 5.273 (d) |
| 34 | Xylose | 3.52 (dd); 3.68 (dd); 3.85 (dd); 4.00 (ddd); 4.585 (d) |
| 35 | Uracil | 5.797 (d); 7.536 (d) |
| 36 | Fumarate | 6.526 (s) |
| 37 | Tyrosine | 3.02 (dd); 3.17 (dd); 3.98 (t); 6.893 (d); 6.93 (ddd); 7.185 (d) |
| 38 | 4-Hydroxyphenylacetate | 3.43 (d); 3.51 (d); 6.879(d); 6.748(d); 6.803 (s); 7.245 (t) |
| 39 | Phenylalanine | 2.84 (dd); 3.27 (dd); 3.98 (t); 7.28 (m); 7.324 (m); 7.378 (m) |
| 40 | Formate | 8.46 (s) |
| 41 | Xanthurenate | 6.9 (s); 7.207 (d); 7.416 (d); 7.725 (d) |

Key: s: singlet; d: doublet; dd: double doublet; ddd: double of doublets of doublets; dtd: doublet of triplet of doublets; t: triplet; q: quartet; m: multiplet
